# Supplementary material for: Sheep genome functional annotation reveals proximal regulatory elements contributed to the evolution of modern breeds
Source: Nat Commun. 2018 Feb 28;9:859. doi: 10.1038/s41467-017-02809-1 (PMC5830443; doi:10.1038/s41467-017-02809-1)
Supplement: Supplementary file 1 — Supplementary Information [file 41467_2017_2809_MOESM1_ESM.pdf]

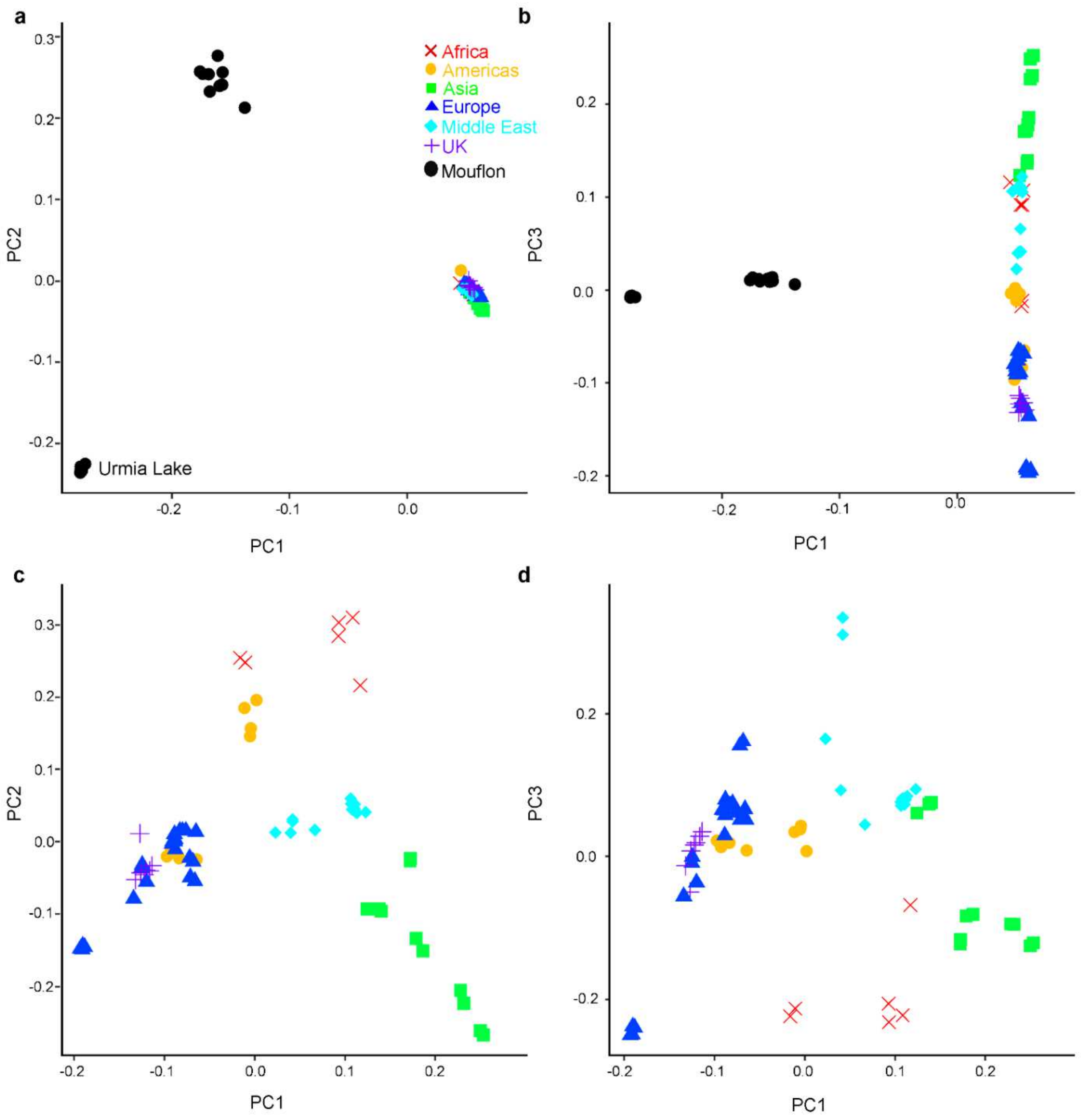

Supplementary Figure 1 | Genomic relationship between individual animals using PCA of pairwise genetic distance. PCA was performed using PLINK v1.90 on a SNP subset identified by LD pruning across genome bins (--indep-pairwise 500 5 0.3). **(a)** All animals plotted for PC1 (6.2%) and PC2 (3.2% of variation). Domestic sheep cluster together and separate from two distinct mouflon populations representing distinct locations. **(b)** All animals plotted for PC1 and PC3 (2.5% variation), capturing variation between domestic sheep genomes. **(c)** Analysis of domestic sheep alone using PC1 (2.5 %) and PC2 (1.8% variation) more clearly demonstrates phylogeographic structure. **(d)** Analysis of domestic sheep alone using PC1 (2.5 %) and PC3 (1.6%).

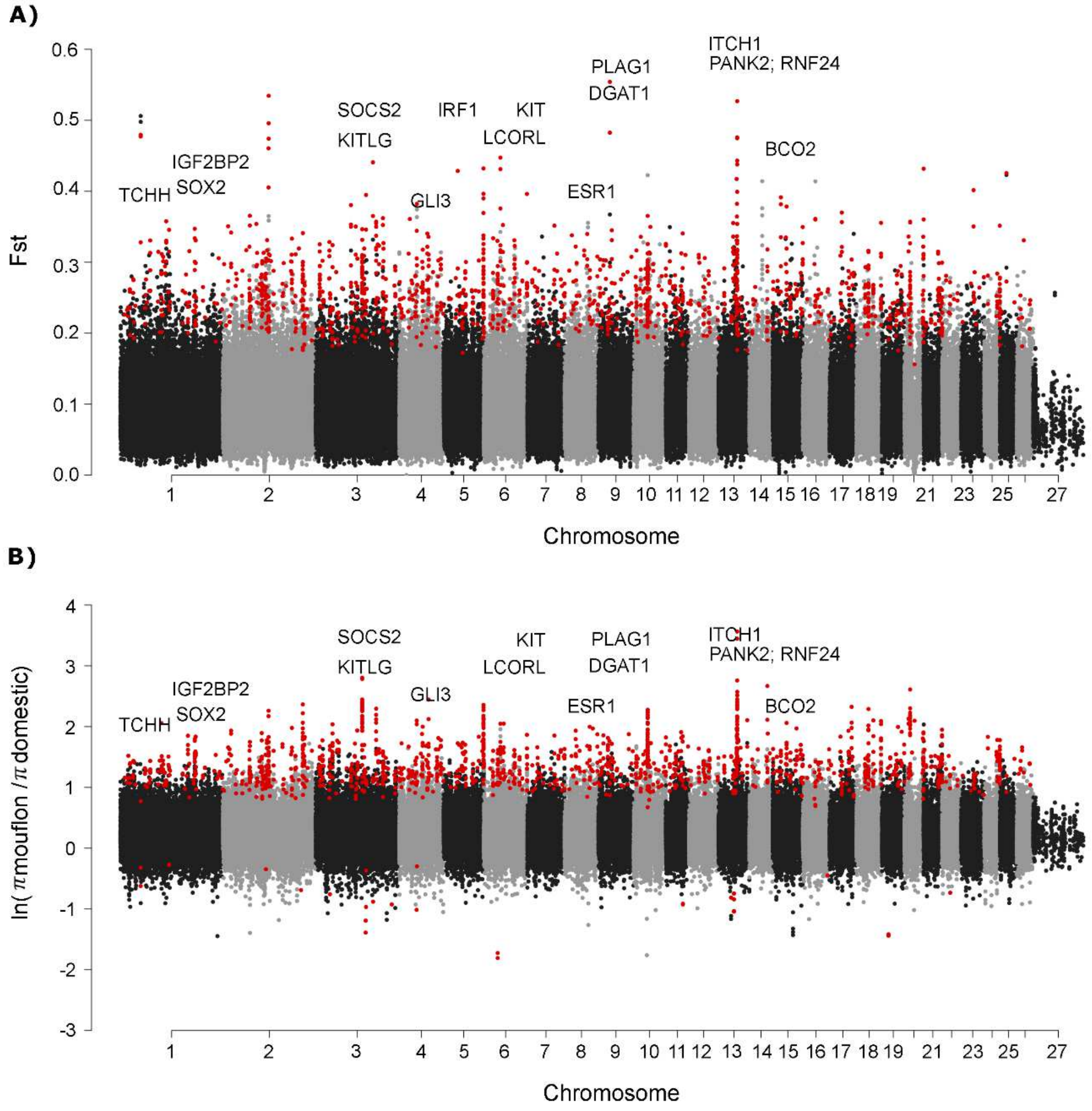

Supplementary Figure 2 | Genome-wide distribution of two selection sweeps metrics. **(a)** F_{ST} comparing domestic and wild sheep genomes averaged in 20 kb genomic bins and plotted in genomic order. Bins highlighted in red are identified as outliers likely to be enriched for selection sweeps after joint consideration of both metrics (refer to Figure 2 and methods). **(b)** Log difference in nucleotide diversity between species. Positive values represent 20 kb genomic bins with depressed nucleotide diversity in 67 domestic sheep genomes compared with 17 wild Mouflon. Genes are identified that have been implicated previously in studies of domestication or variation in production traits.

a

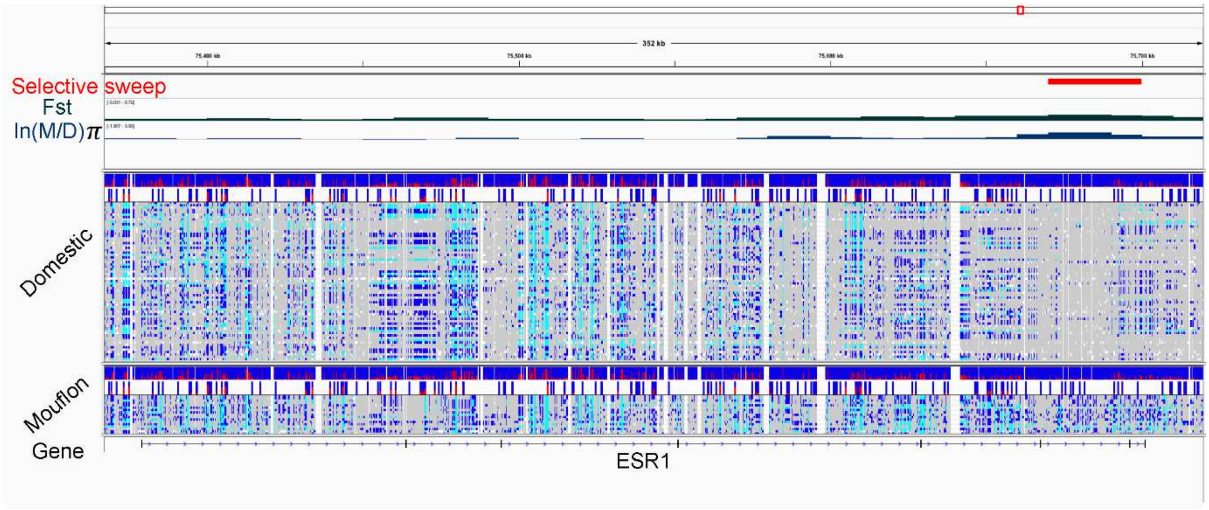

b

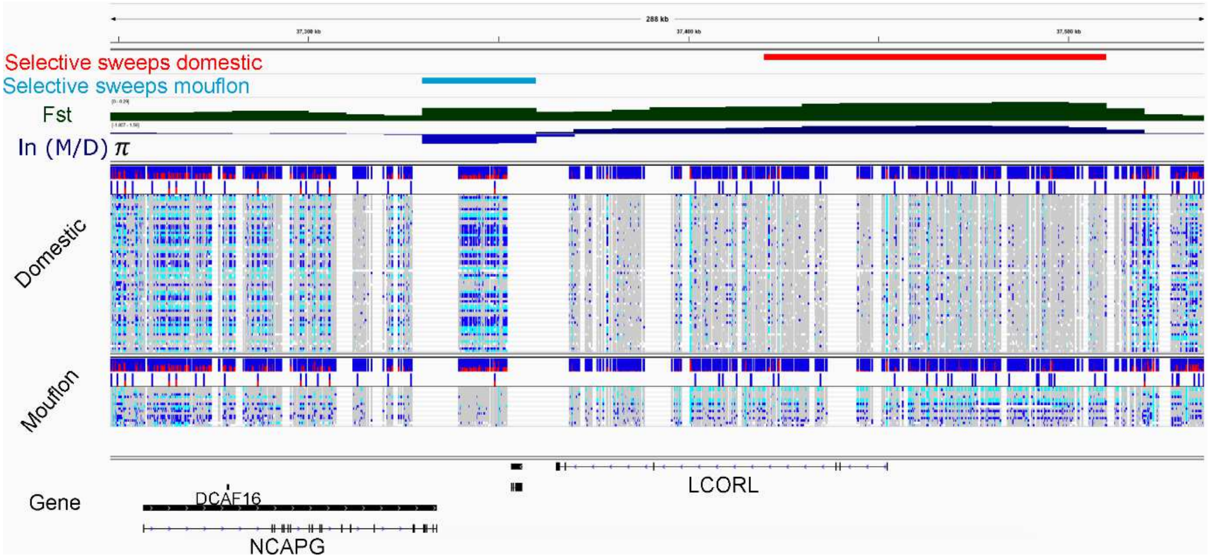

Supplementary Figure 3 | Illustration of two sweep regions. (a) The integrative genome viewer (IGV) was used to plot a 350 kb region of sheep chromosome 8 containing *ESR1*. The genotypic status for each genome is given as heterozygous (dark blue) or homozygous for the reference (grey) or alternate allele (light blue). Annotation tracks highlight the outlier region (red bar) arising from joint consideration of the two selection sweep metrics (black and blue bars). The location of *ESR1* exons is given at the bottom. Note the sweep is characterized by a lack of variability amongst domestic sheep genomes. **(b)** IGV plot spanning 288 kb of chromosome 6 (Mb 37.2 – 37.5) containing *NCAPG* and *LCORL*. The region exhibits evidence of selection within both domestic sheep (red) and wild mouflon (aqua-blue).

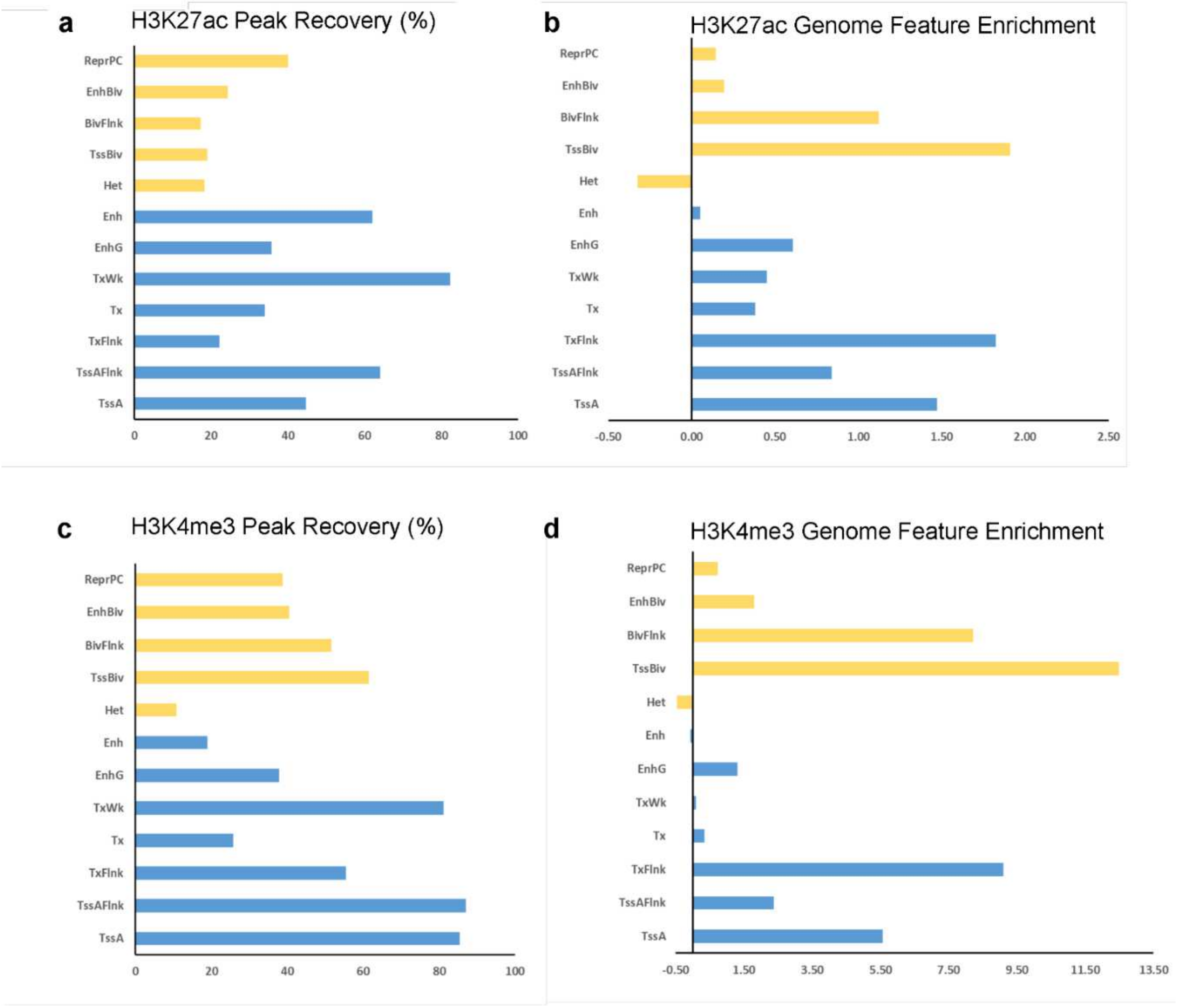

Supplementary Figure 4 | Recovery of experimentally defined sheep promoters and enhancers in predicted epigenome features. (a) ChIP-seq analysis of sheep adipose tissue using H3K27ac identified 35622 peaks enriched for active promotors and enhancers. The percentage peak recovery within each of 12 predicted epigenome marks is shown. **(b)** Given the predicted epigenome marks span a variable amount of the sheep genome, fold enrichment analysis was performed following 1000 randomizations. Increasingly positive values indicate elevated recovery beyond expectation. Epigenome features predicted from human Epigenome Roadmap active (blue) and repressive (yellow) chromatin states are coloured separately. **(c)** ChIP-seq analysis of sheep adipose tissue using H3K4me3 identified 16098 peaks tagging active promotor elements. Recovery of predicted epigenome features is shown, along with **(d)** fold enrichment for promoters estimated 1000 randomizations.

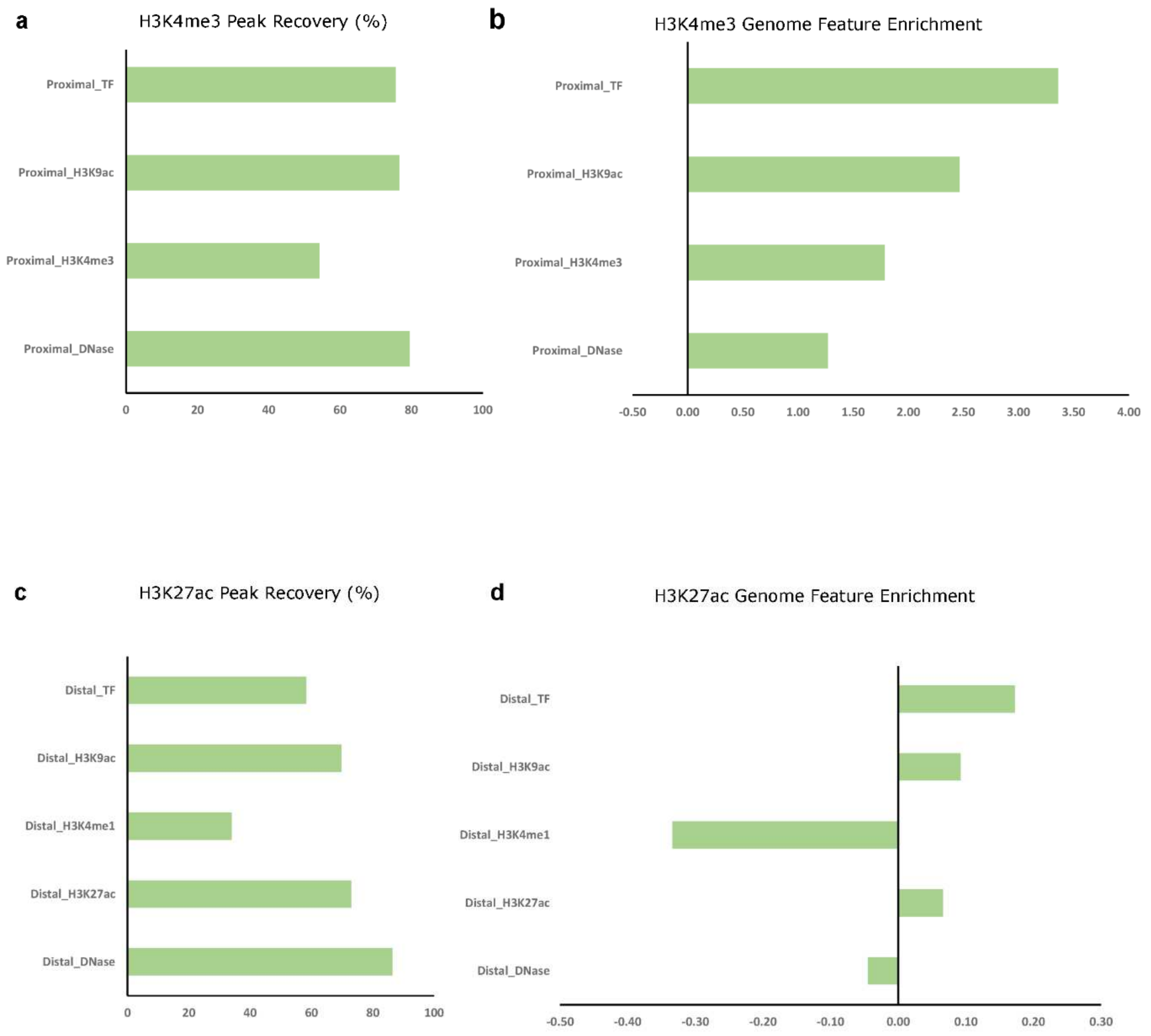

Supplementary Figure 5 | Recovery of experimentally defined sheep promoters and enhancers in predicted distal and proximal genome features. Predicted proximal genome features from ENCODE cell lines assessed against experimentally obtained sheep promoters using H3K4me3 ChiP-seq (16098 peaks) **(a,b)** and predicted distal genome features from ENCODE cell lines were assessed against experimentally obtained sheep enhancers (H3K27ac peaks – H3K4me3 peaks by ChiP-seq; 26000 peaks) (**c,d**). (**a**) The proportion of H3K4me3 overlapping with each genome feature is given as a percentage. (**b**) Fold enrichment was estimated following 1000 randomizations to establish the number of overlaps expected by change. **(c)** The proportion of H3K27ac with no H3K4me3 signal (enhancers) overlapping with each genome feature is given as a percentage. (**d**) Fold enrichment was estimated following 1000 randomizations to establish the number of overlaps expected by chance.

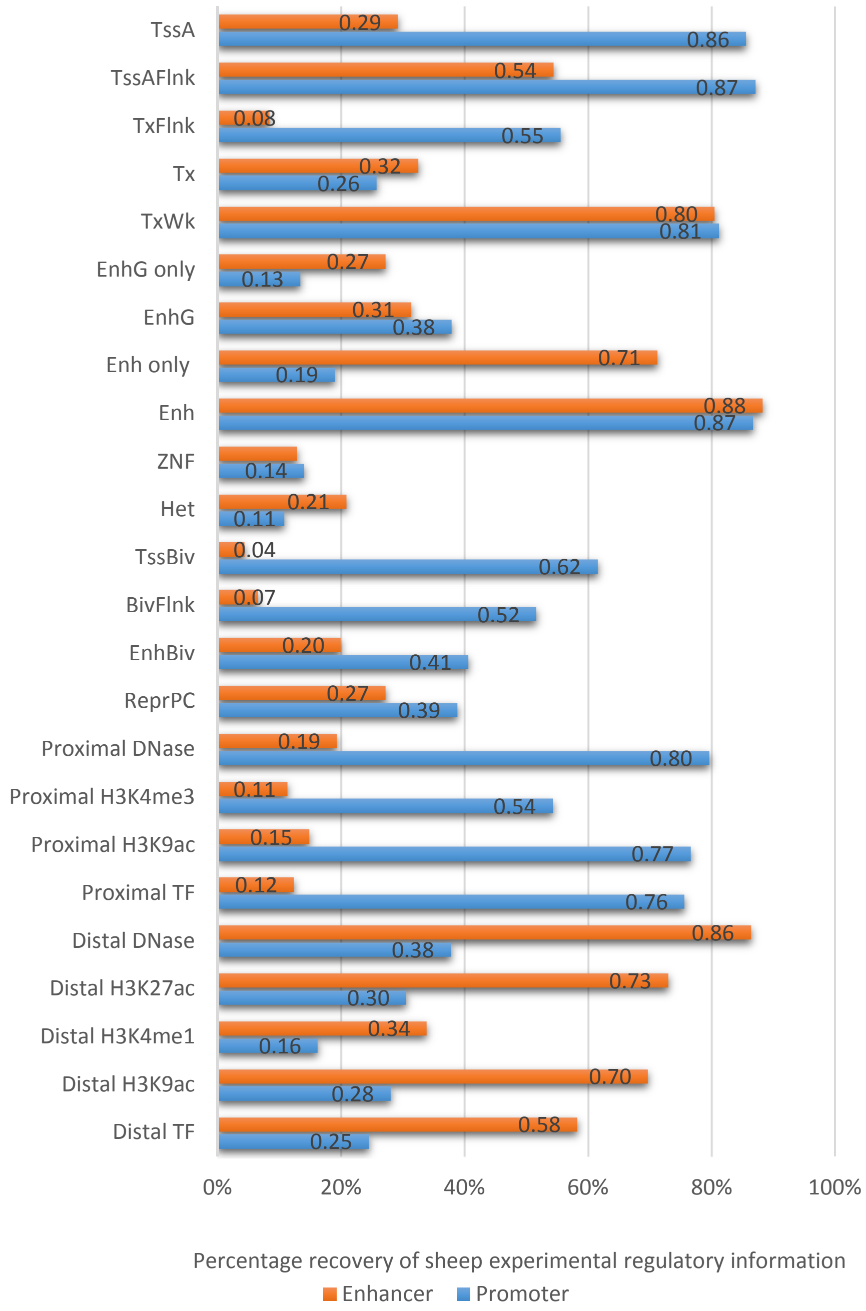

Supplementary Figure 6 | Validation of predicted regulatory features using ChIP-Seq data. The availability of experimentally derived H3K4me3 histone modification (marking proximal promoters), and H3K27ac (marking both enhancers and promoters), allows us to distinguish between these two categories of regulatory elements. We define promotors (blue) as peaks identified using H3K4me3. We define enhancers (orange) as H3K27ac peaks not identified by H3K4me3. This approach was also applied to the predicted genome features. The ‘Enh only’ predicted feature represents the Roadmap Enhancers ‘Enh’, following subtraction of promotor regions identified as ‘TssA’. The recovery of each predicted genome features is shown separately within our experimentally derived promotor and enhancers.

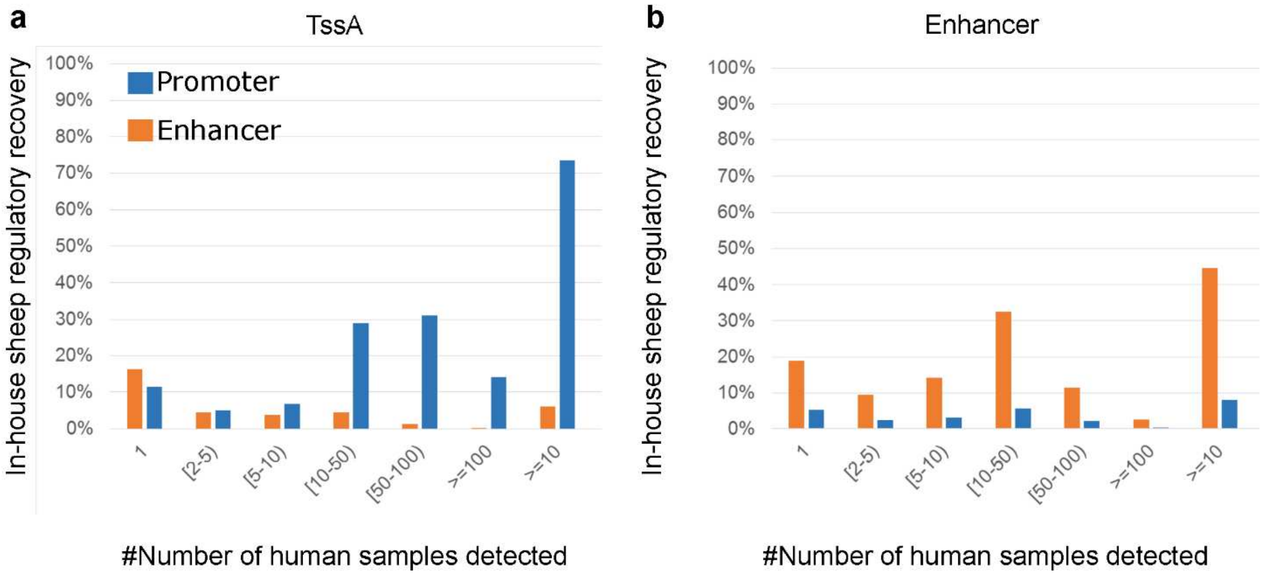

Supplementary Figure 7 | Validation of sheep promoter (blue) and enhancer (orange) recovery from (a) chromHMM TssA mark, based on the number of human samples detected (tissue-specificity) **(b)** Predicted enhancers based on RoadMap chromHMM excluding enhancer overlapping chroHMM TssA, based on the number of human samples detected (tissue-specificity).

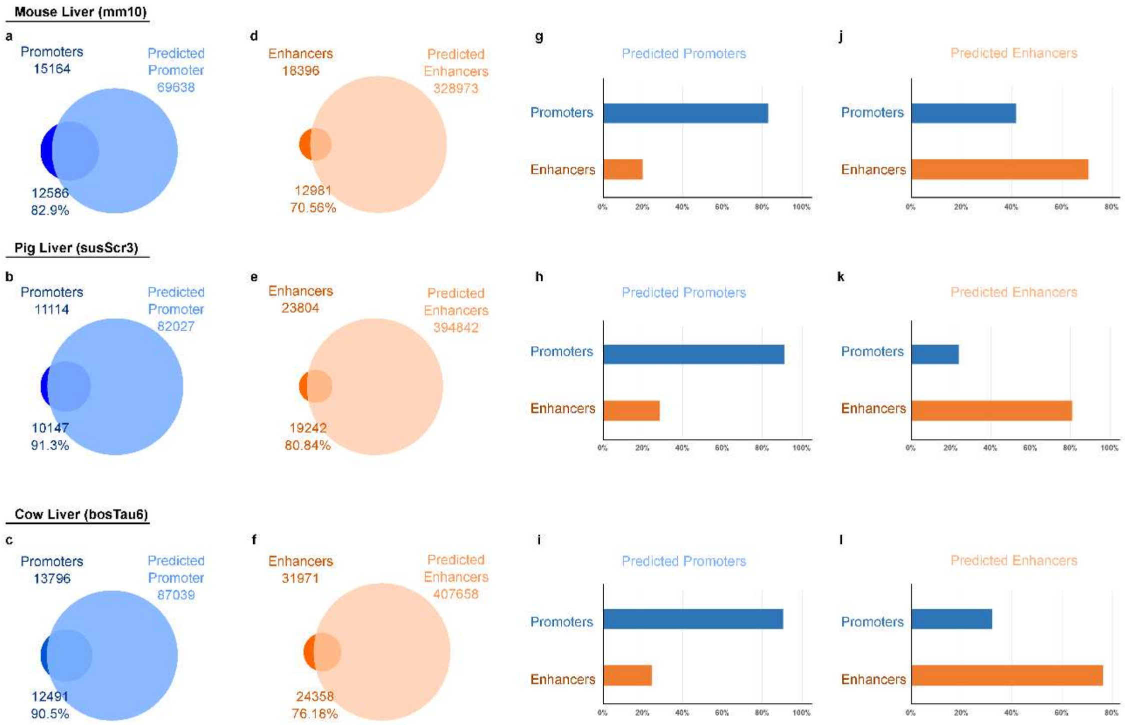

Supplementary Figure 8 | Robustness in the inference of homologous regions between assemblies using the liftOver UCSC tool [1]. The comparative approach used in this study employs liftOver genome coordinates conversion. This is based on chain and net files between genome assemblies. Chains refer to ordered sequence of pairwise nucleotide alignments by BLASTZ 2 separated by large gaps. Nets are a higher-order alignments, which track which bases are covered by more than one chain as well as annotating inversions and or other displacements between genome assemblies. Nets are not symmetric, meaning they show the best alignment of one species versus the other with the consequence they can differ in the reciprocal net. To optimise for the best alignment from both species we prepare a “reciprocal best” alignment. It is noteworthy that this method does not allow us to identify lineage-specific duplications. It is important to evaluate if this approach is overly sensitive to the particular combination of species in which it is applied. We therefore tested our liftOver pipeline to convert human Roadmap Epigenomics chromHMM TssA and Enh data into **(a)** mouse (mm10), **(b)** pig (susScr3) and **(c)** cow (bosTau6). The predicted functional elements in each genome were then

assessed for enrichment within species specific experimentally derived H3K4me3 regions (promoters) and enhancers (regions with H3K27ac signal not overlapping H3K4me3 regions)[17]. This revealed that, as in sheep, liftOver of human promoters and enhancers from 127 epigenomes successfully recovered high proportions of mouse, pig and cow functional elements. Next, as a proxy to obtain true positive (TPR) and true negative rate (TNR) estimates in our predictions we present the proportion of experimentally identified liver promoters and enhancers captured by the predicted promoters pipeline across different species **(g)** mouse **(h)** pig and **(i)** cow, and finally, we present as TPR the proportion of experimentally identified liver enhancers in the predicted enhancers pipeline and as TNR the proportion of liver promoters in **(j)** mouse **(k)** pig in **(l)** cow.

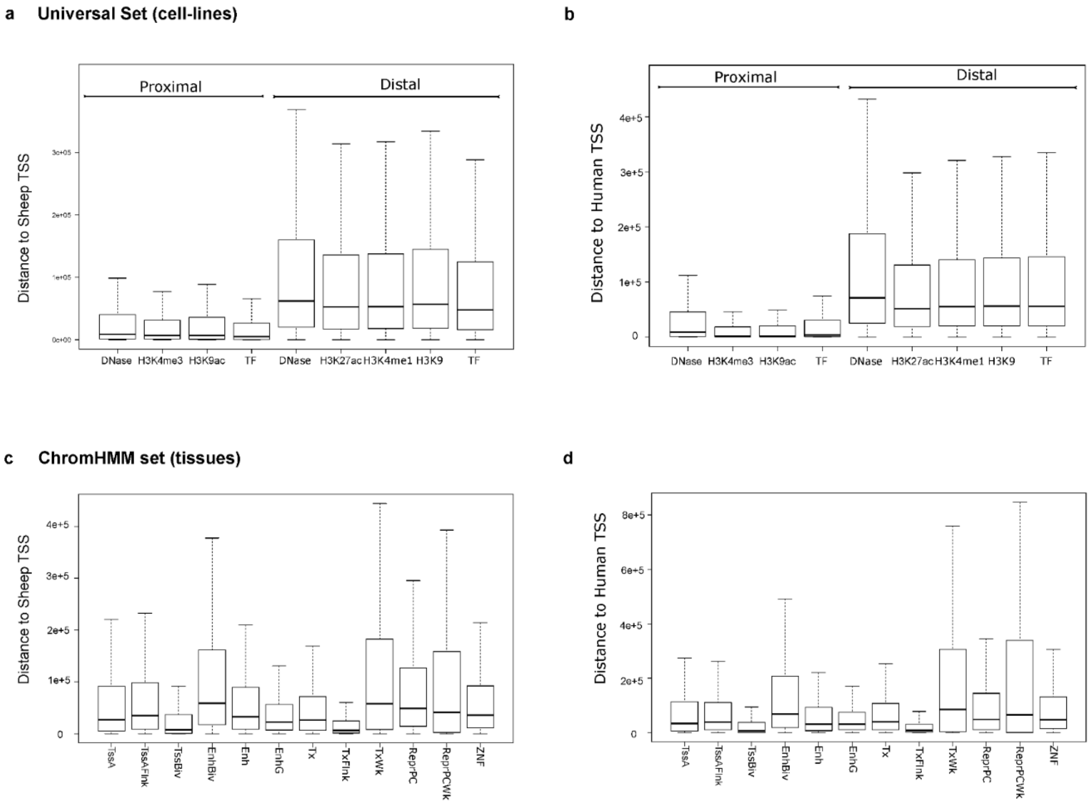

Supplementary Figure 9 | Physical proximity of predicted genome features to transcription start sites in the human and sheep genomes. (a) The distance to sheep TSS is shown for each of nine genome features predicted from ENCODE data. Predicted proximal features are consistently closer than distal ones. **(b)** Distance to human TSS for each predicted ENCODE genome feature. **(c)** The distance to sheep TSS is shown for each of 12 chromatin states predicted from Roadmap Epigenome data. **(d)** Distance to human TSS for each predicted Roadmap genome feature. Boxplots represent the distance of predicted genome features to TSSs through their quartiles. The bottom and top of the box represents the first and third quartiles. The band in the middle the median or second quartile. The ends of the whiskers represent the lowest datum within 1.5 of the interquartile range of the lower quartile and the highest datum within the 1.5 inter quartile of the upper quartile.

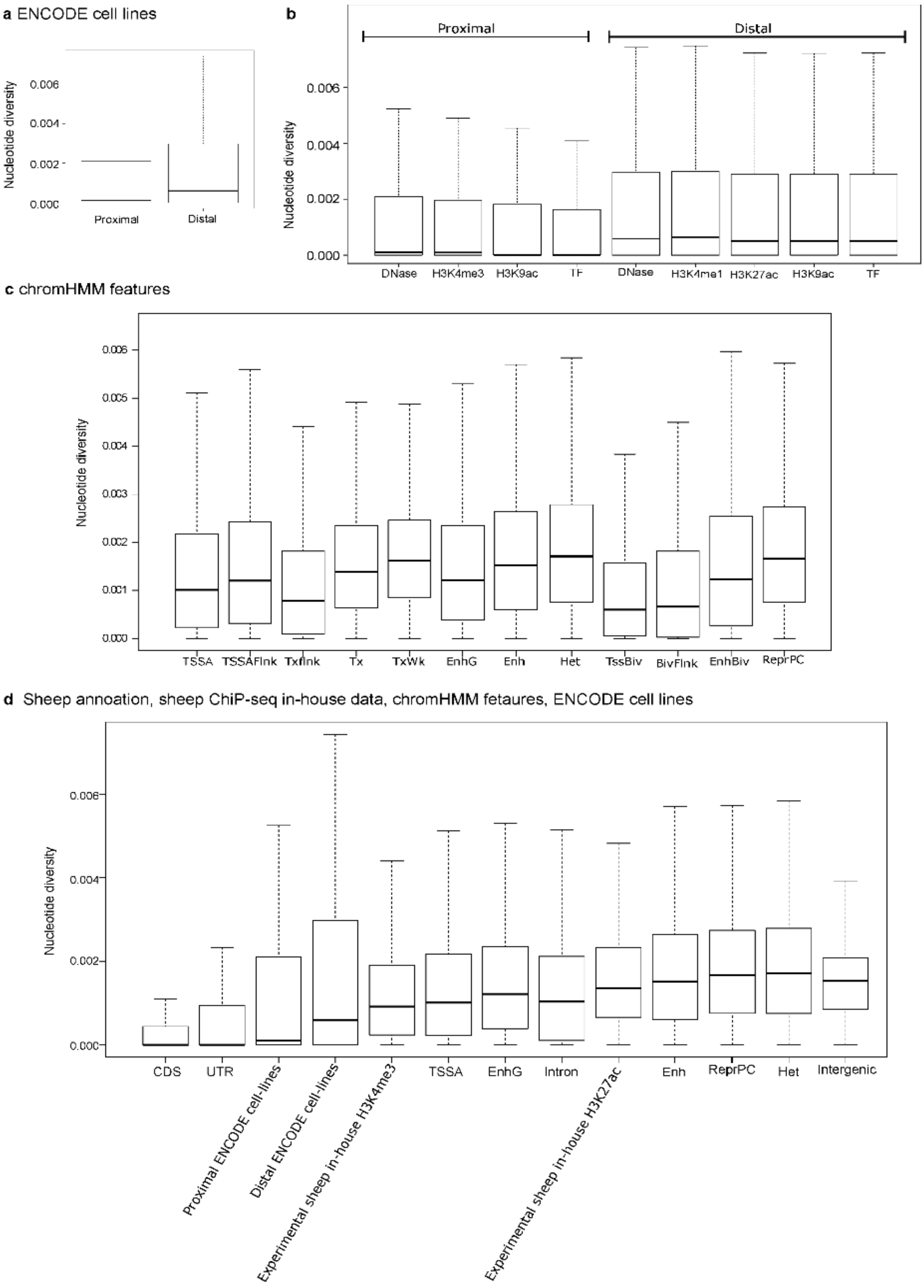
a ENCODE cell lines
Nucleotide diversity
0.000
0.002
0.004
0.006
Proximal
Distal
b
Proximal
Distal
Nucleotide diversity
0.000
0.002
0.004
0.006
DNase
H3K4me3
H3K9ac
TF
DNase
H3K4me1
H3K27ac
H3K9ac
TF
c chromHMM features
Nucleotide diversity
0.000
0.001
0.002
0.003
0.004
0.005
0.006
TSSA
TSSAFlnk
Txflnk
Tx
TxWk
EnhG
Enh
Het
TssBiv
BivFlnk
EnhBiv
ReprPC
d Sheep annoation, sheep ChiP-seq in-house data, chromHMM fetaures, ENCODE cell lines
Nucleotide diversity
0.000
0.002
0.004
0.006
CDS
UTR
Proximal ENCODE cell-lines
Distal ENCODE cell-lines
Experimental sheep in-house H3K4me3
TSSA
EnhG
Intron
Experimental sheep in-house H3K27ac
Enh
ReprPC
Het
Intergenic

Supplementary Figure 10 | Nucleotide diversity compared between predicted genome features. **(a)** Higher nucleotide diversity was observed within distal predicted elements expected to be less evolutionarily constrained than proximal regulatory features. **(b)** Nucleotide diversity is plotted separately for each proximal and distal epigenome mark predicted from the ENCODE dataset. **(c)** Nucleotide diversity is compared across predicted chromatin states from the Roadmap dataset. **(d)** Nucleotide diversity compared across both experimentally derived genome features and from the reference assembly OARv3.1. Boxplots represent the distribution of nucleotide diversity per genome feature through their quartiles. The bottom and top of the box represents the first and third quartiles. The band in the middle the median or second quartile. The ends of the whiskers represent the lowest datum within 1.5 of the interquartile range of the lower quartile and the highest datum within the 1.5 inter quartile of the upper quartile.

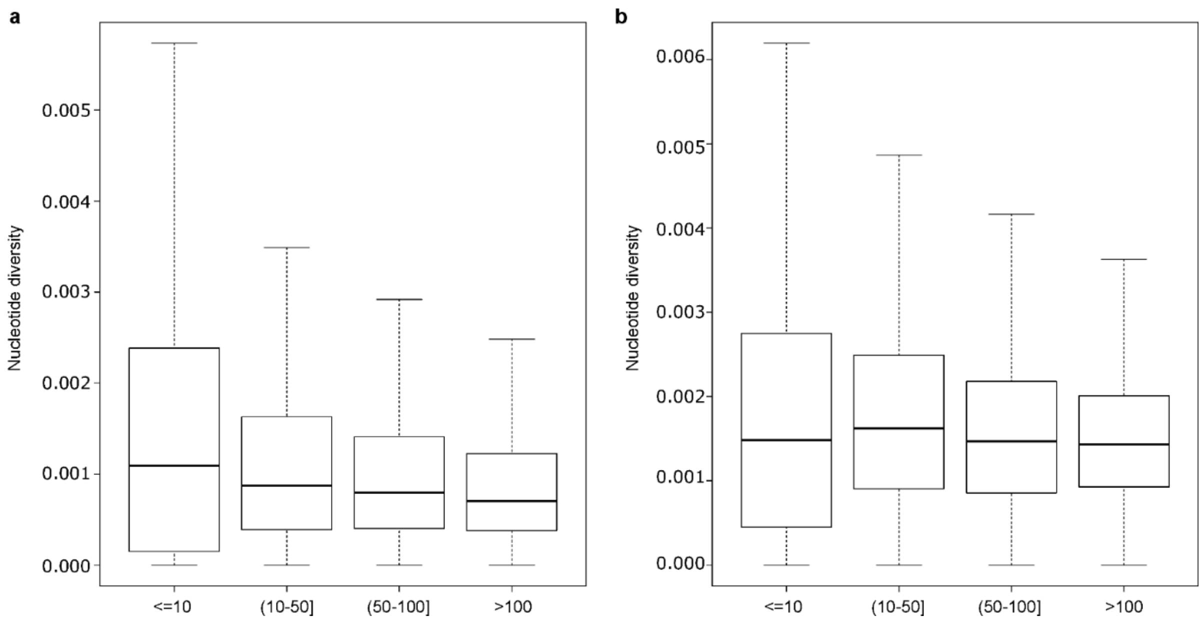

Supplementary Figure 11 | Decreasing nucleotide diversity in elements ubiquitously active (>100 tissues). Nucleotide diversity in domestic sheep population for **(a)** TSSAs and **(b)** enhancers detected in different number of tissues. Boxplots represent the distribution of nucleotide diversity per feature through their quartiles. The bottom and top of the box represents the first and third quartiles. The band in the middle the median or second quartile. Finally the ends of the whiskers represent the lowest datum within 1.5 of the interquartile range of the lower quartile and the highest datum within the 1.5 inter quartile of the upper quartile.

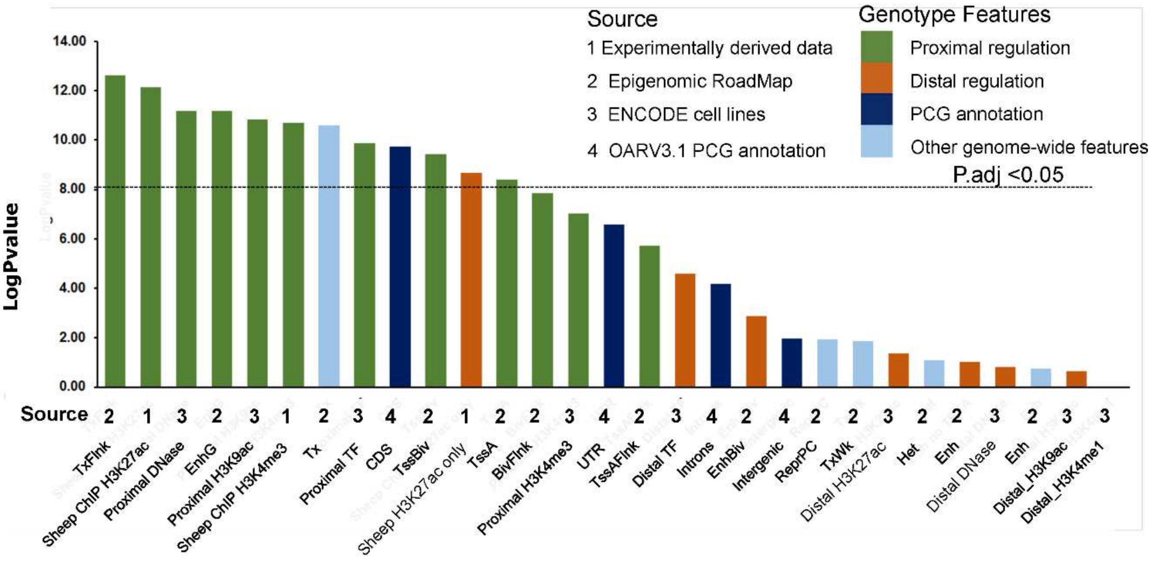

Supplementary Figure 12 | Strength of genome feature enrichment within 635 sweep regions. Overlap between sweep regions and annotated genomic features was performed using LOLA [1]. Genome features originate from four sources (numbered) and can be classified as broadly proximal, distal, describing protein coding genes or other (colour coded). The 12 genome features significantly enriched mainly represent proximal components of the gene regulatory machinery, along with coding exons and sites actively transcribed.

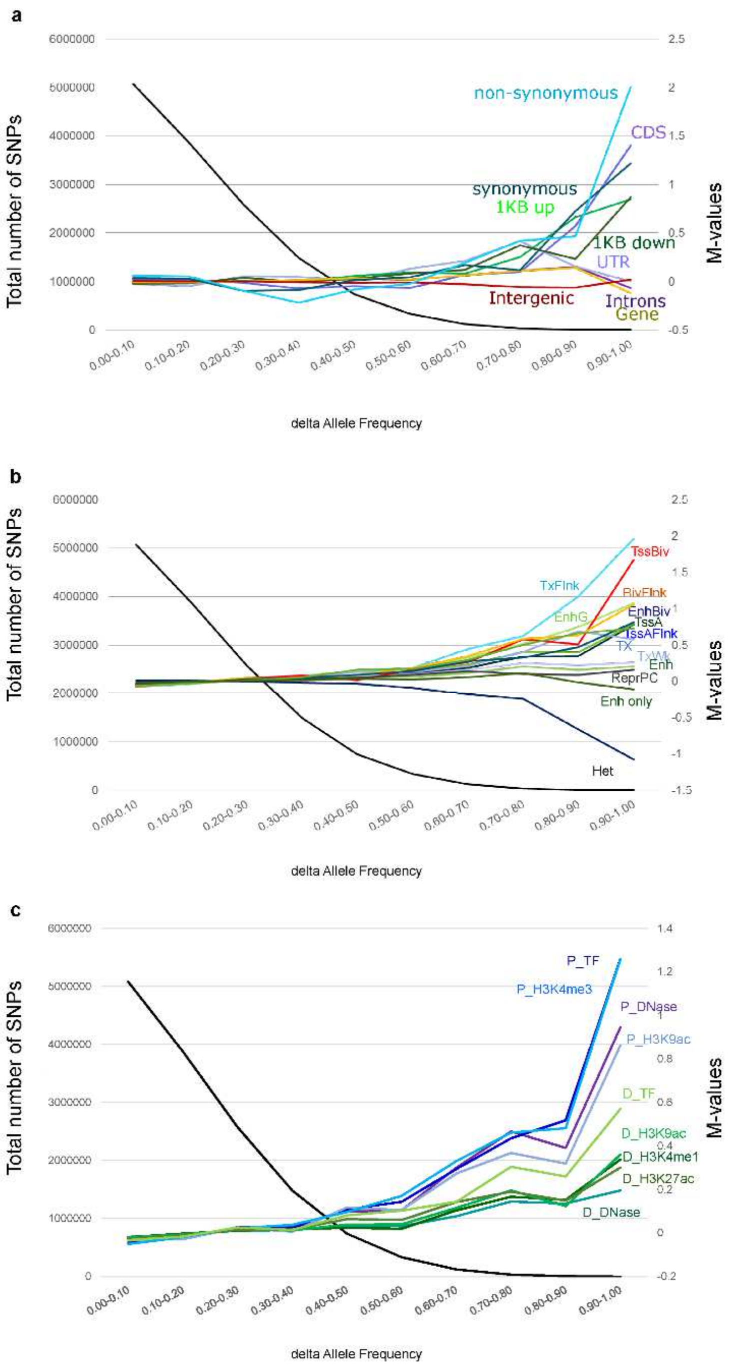

Supplementary Figure 13 | Delta allele frequency (dAF) for SNPs residing in annotated genome features. dAF calculated for two populations domestic *vs* mouflon. The black line represents the total number of SNPs in each dAF bin and coloured lines represent M values of different SNP types. M values were calculated by comparing the frequency of SNPs in a given annotation category in a specific bin with the corresponding frequency across all bins. **(a)** Genomic features based on current sheep annotation (v.1.81) **(b)** chromHMM features translated to sheep coordinates **(c)** ENCODE cell line universal set translated to sheep coordinates.

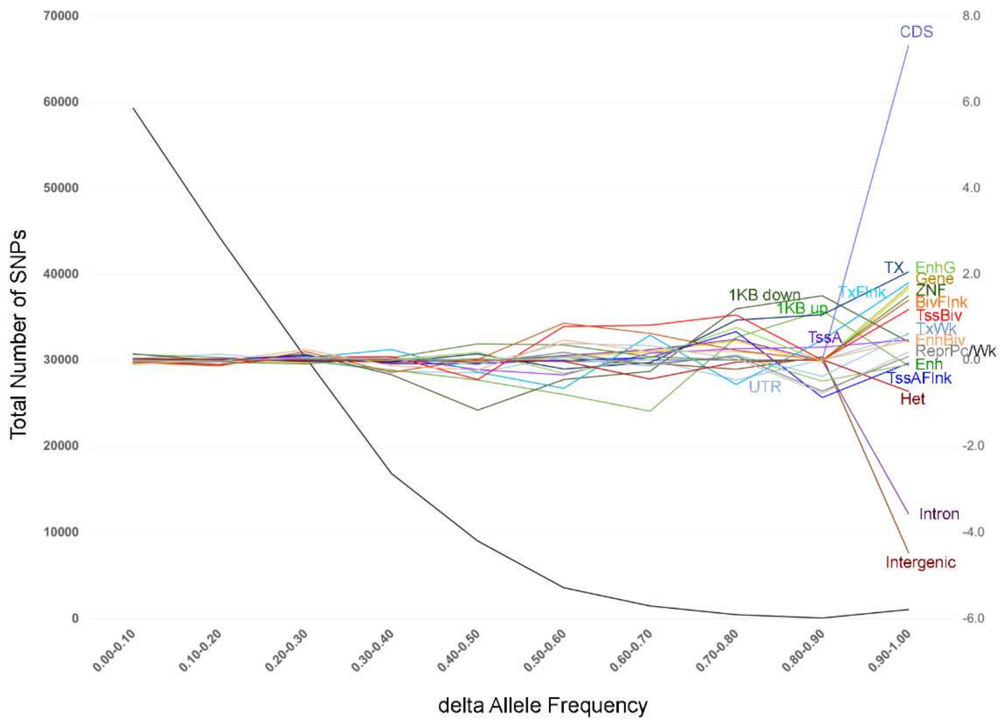

Supplementary Figure 14 | Allele frequency differences (ΔAF) between wild and domestic sheep following artificial manipulation of exons. To evaluate correlated effects between annotation features, exonic SNP were artificially altered to take extreme values (ΔAF = 0.95, which is in the highest bin) in a random set of 1420 20 Kb bins. The M-value reflects the strength of enrichment beyond expectation (refer to methods) and the black line gives the total number of SNPs in each ΔAF bin. Protein coding gene annotations are plotted along with 12 chromatin state annotations from the Roadmap dataset.

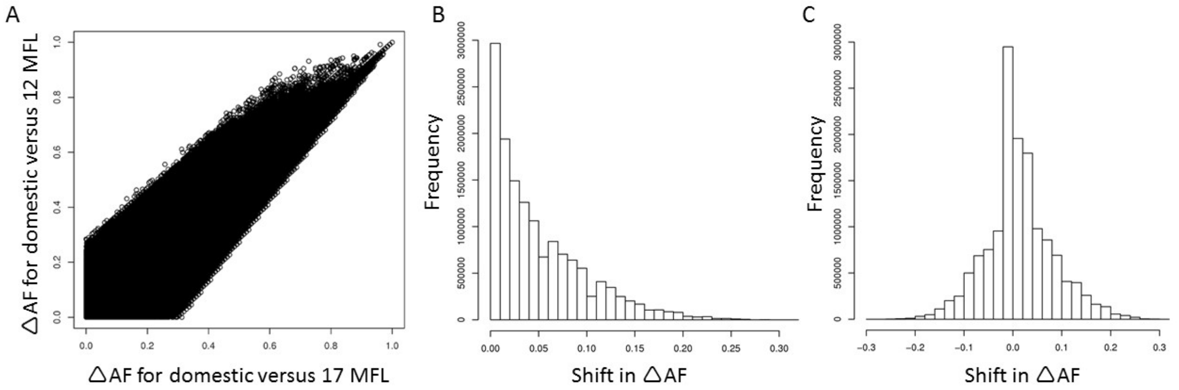

Supplementary Figure 15 | Allele frequency difference comparison. The sampling of domestic and wild genomes is likely to have some influence on estimates of diversity and the detection of selection sweeps. We evaluated the impact of reducing the number of Mouflon genomes included in the estimation of observed allele frequency differences. Five Mouflon were selected for removal based on their membership of a population subgroup (Urmia Lake, Supplementary Figure 1) that are likely highly inbred. Allele frequency difference (△AF) was assessed as it is the basis for the site frequency testing and relates to selection sweep methodology. The correlation of allele frequencies was high (r^2 = 0.96) when estimated using all available Mouflon (n = 17) or a reduced number (n = 12). (**a**) Allele frequency difference between domestic sheep and mouflon was compared using all Mouflon or the smaller subset (r^2 = 0.87). This indicates that the shift in △AF between species was not large in response to removal of the Mouflon genomes. (**b**,**c**) The magnitude in △AF shift is given as histograms, and shows that few loci have values exceeding 0.3. This indicates the use of a reduced representation of Mouflon has a measurable impact that is unlikely to influence the major findings of the study.

Supplementary Table 1: Distribution of Consequences types across domestic and Mouflon. 60.1% of the variants are intergenic regions and 30.4% introns.

Consequence Type	Counts
splice_acceptor_variant	417
splice_donor_variant	413
stop_gained	1,211
stop_lost	61
initiator_codon_variant	205
missense_variant	84,871
splice_region_variant	23,916
stop_retained_variant	87
synonymous_variant	133,891
coding_sequence_variant	83
mature_miRNA_variant	168
5_prime_UTR_variant	9,340
3_prime_UTR_variant	63,868
non_coding_transcript_exon_variant	7,294
intron_variant	9,196,042
non_coding_transcript_variant	7,568
upstream_gene_variant	1,261,312
downstream_gene_variant	1,250,954
intergenic_variant	17,989,136

Supplementary Table 2: Distribution of Coding Consequences types across domestic and Mouflon sheep

Consequence Type	Counts
stop_gained	1,211
stop_lost	61
missense_variant	84,871
synonymous_variant	133,891
coding_sequence_variant	83

Supplementary Table 3: Variants Summary statistics per phylo-geographic group and individual

Region	Reference	Het	Alternative	Total
**Africa**	21261190	3575177	2335157	27171523
**Americas**	20788745	3902309	2058070	26749124
**Asia**	21087868	3408676	2368399	26864943
**Europe**	21138054	3788680	2020560	26969705
**Middle East**	20974220	3788680	2180280	269431
**Mouflon**	20461963	4142895	3183907	27788764
**United Kingdom**	20419443	3882973	1834499	26136915

Supplementary Table 4: Genes associated with selective sweeps in domestic animals reported in other studies.

Chr	Start	End	Avg rnk Fst & Pi	Pi	Fst	Closest Genes	Distance to closest gene	Function	Ref
13	50310001	50650000	2	3.56	0.53	*PANK2*	0	Neurodegeneration	2,3
3	124790001	125080000	21.5	2.80	0.35	*KITLG*	27570	Coat colour	4–6
2	184990001	185040000	42.5	1.98	0.33	*GLI2*	139099	Growth	7
1	199640001	199790000	51	1.79	0.35	*IGF2BP2*	0	Adiposity	8–10
4	78890001	79000000	51.5	2.44	0.31	*GLI3*	29289	Pigmentation	11
17	59450001	59520000	62.5	2.32	0.30	*TBX3*	215541	Pigmentation	12
9	36140001	36240000	63	1.72	0.34	*PLAG1*	0	Fertility, stature	13–16
15	21900001	22010000	70.5	1.60	0.39	*BCO2*	0	Yellow-fat	17–19
9	13570001	13610000	82.5	1.85	0.30	*DGAT1*	0	Milk fat	20,21
6	37420001	37510000	144	1.56	0.29	*LCORL*	0	Weight/height	22–25
3	124640001	124680000	184	1.69	0.26	*KITLG*	0	Coat colour	4–6
8	75670001	75700000	228.5	1.97	0.24	*ESR1*	0	Litter size; prolifacy	26,27
6	38500001	38530000	245.5	1.62	0.25	*LCORL*	1047670	Weight/height	22–25
5	19460001	19480000	312.5	1.23	0.27	*IRF1*	5040	Immune function	28,29
3	129730001	129750000	359	0.94	0.31	*SOCS2*	7494	Weight and milk production	30
1	100990001	101010000	383.5	1.26	0.24	*TCHH*	0	Hair	31,32
4	78800001	78820000	386.5	1.15	0.26	*GLI3*	209289	pigmentation	11
5	85880001	85900000	405	1.11	0.26	*MEF2C*	173852	Skeletal muscle development	28,33,34
2	105830001	105850000	417.5	1.27	0.23	*HAND2*	235173	Limb development	35
20	17530001	17550000	434.5	1.207	0.24	*VEGFA*	147890	Reproduction	36,37
13	63380001	63400000	469	1.237	0.22	*ITCH*	0	Coat colour	38
1	203460001	203480000	481	0.977	0.25	*SOX2*	52282	Stem cell maintenance	39
6	35290001	35310000	486.5	1.05	0.24	*GPRIN3*	201294	Brain development	40
6	70290001	70310000	492.5	1.24	0.21	*KIT*	55390	Coat colour	41,42

Supplementary Table 5: Summary statistics on regulatory regions from the ROADMAP dataset, universal set (cell lines) and chromHMM features (human 127 tissues epigenomes) reciprocal liftOver to sheep genome (minMatch=0.1) oarv3.1.

		Human					Sheep					
chromHMM		# Features	# Merged Features	Average bp	Total bp	% human genome	# Features sheep	% Recovery	# Merged	Average bp	Total bp	% Sheep genome
Active TSS	TSSA	2525249	146860	1053	154645000	4.93	1036207	41.03	82792	995	82359149	3.18
Flanking active TSS	TSSAFlnk	3652921	294595	935	275342400	8.78	1773713	48.56	183766	816	149940350	5.79
Transcribed at 5' and 3' UTR	TxFlnk	277589	61004	1183	72138400	2.30	111873	40.30	34251	936	32067982	1.24
Strong Transcription	Tx	3883996	94165	6101	574463800	18.31	721092	18.57	89599	2526	226333266	8.75
Weak Transcription	TxWk	10396279	127882	15776	2017410200	64.31	1914957	18.42	201299	3580	720684948	27.85
Genic Enhancers	EnhG	1302269	157928	1829	288851800	9.21	464496	35.67	100036	1211	121183647	4.68
Enhancers	Enh	11214553	553599	2234	1236774800	39.42	4384817	39.10	415857	1413	587677260	22.71
Heterochromatin	Het	4071861	232924	5257	1224371800	39.03	289704	7.11	119185	1449	172657767	6.67
Bivalent /Poised Enhancers	TssBiv	496055	45889	1071	49154400	1.57	223409	45.04	27773	1022	28391816	1.10
Flanking Bivalent TSS /Enh	BivFlnk	649112	59187	704	41680400	1.33	327143	50.40	37351	670	25012932	0.97
Bivalent Enhancer	EnhBiv	1101156	165361	896	148152400	4.72	548188	49.78	98442	786	77370235	2.99

Repressed Polycomb	ReprPC	2434414	199871	3339	667283800	21.27	753572	30.95	119952	1881	225672647	8.72
Universal set												
Proximal_DNase		607894	607894	150	91358400	2.91	347343	57.14	341731	156	53394400	2.06
Proximal_H3K4me3		4256692	420127	150	63159010	2.01	1810021	42.52	166587	156	25926035	1.00
Proximal_H3K9ac		1413622	326690	150	49118460	1.57	863085	61.05	193180	156	30047479	1.16
Proximal_TF		384343	384343	150	57786950	1.84	147963	38.50	145993	156	22717165	0.88
Distal_DNase		2558595	2558595	150	384123670	12.24	1397839	54.63	1373967	159	217776308	8.42
Distal_H3K27ac		3557364	1126338	150	169123020	5.39	2156448	60.62	632067	158	99748144	3.85
Distal_H3K4me1		5675289	1516873	150	227752370	7.26	1634217	28.80	374953	158	59207183	2.29
Distal_H3K9ac		2083470	944139	150	141772490	4.52	1283569	61.61	550604	158	86765982	3.35
Distal_TF		1122364	1122364	150	168531380	5.37	367515	32.74	361496	158	57180633	2.21

Supplementary Table 6: Results on testing the significant overlap between annotated genomic features and ovine adipose tissue histone modification marks compared to 1000 genome-wide randomizations per feature.

Genomic Features	# Original features	Original overlap sheep H3K27ac	Average overlap sheep H3K27ac in permutations	#times bigger than original in
**H3K27ac**				
**TssA**	82792	15760	6376	0
**TssAFlnk**	183766	22579	12267	0
**TxFlnk**	34251	7805	2765	0
**Tx**	89599	11987	8688	0
**TxWk**	201299	76039	46461	0
**EnhG**	100036	12618	7853	0
**Enh**	415857	70793	43829	0
**Enh only**	346131	21891	20808	0
**Het**	119185	6440	9538	1000
**TssBiv**	27773	6672	2291	0
**BivFlnk**	37351	6089	2870	0
**EnhBiv**	98442	8582	7179	0
**ReprPC**	119952	10373	10280	128
**H3K4me3**				
**Proximal_DNase**	341731	12803	5640	0
**Proximal_H3K4me3**	166587	8726	3131.35	0
**Proximal_H3K9ac**	193180	12326	3558.45	0
**Proximal_TF**	145993	12164	2790.99	0
**H3K27ac only**				
**Distal_DNase**	1373967	22871	23939.1	0
**Distal_H3K27ac**	632067	19321	18123	0
**Distal_H3K4me1**	374953	8972	13468.9	0
**Distal_H3K9**	550604	18453	16892.9	0
**Distal_TF**	361496	15427	13159.7	0
**H3K27me3**				
**TssA_no_TssBiv**	82792	2628	3412	1000
**TssAFlnk_no_TssBiv**	183766	5031	7728	1000
**TxFlnk**	34251	1939	2012	951
**Tx**	89599	2615	6677	1000
**TxWk**	201299	12651	16657	1000
**EnhG**	100036	2469	5788	1000
**Enh**	415857	16557	21304	1000
**Enh only**	346131	11187	15720	1000
**Het**	119185	7236	7104	38
**TssBiv**	27773	6820	1675	0
**BivFlnk**	37351	6916	2061.52	0
**EnhBiv**	98442	10739	5194	0
**ReprPC**	119952	14388	7777	0

Supplementary Table 7: Results on testing the significant overlap between annotated genomic features from human Epigenomics Roadmap ,TssA and Enh into mouse (mm10), pig (susScr3) and cow (bosTau6) genome annotations and each species specific H3K27ac regions from public dataset [43] E-MTAB-2633.

Genomic Features	# Original features	Original overlap species H3K27ac	Average overlap sheep H3K27ac in permutations	#times bigger than original in
**H3K27ac mm10**				
**TssA**	69638	14338	3128	0
**Enh**	328973	18428	14323	0
**H3K27ac susScr3**				
**TssA**	82027	16212	4431	0
**Enh**	394842	24539	19372	0
**H3K27ac bosTau6**				
**TssA**	87039	20378	4902	0
**Enh**	407658	29165	23940	0

Supplementary Table 8: Overlap of domestic selective sweeps (1420 regions of 20 kb) regions and list of annotated genomic features based on LOLA [1].

Genomic Feature	Log p-val	Log Odd ratio	support	RnK V	Rnk LO	Rnk Sup	Max Rnk	Mean Rnk	b	c	d	Size
EnhG	12.83	1.50	552	2	3	21	21	8.67	72240	868	170678	100036
Tx	10.68	1.44	573	4	6	19	19	9.67	77654	847	165264	89599
TxFlnk	8.03	1.48	280	5	4	46	46	18.3	34521	1140	208397	34251
10KB Upstream	6.28	1.37	340	6	10	39	39	18.3	45387	1080	197531	20921
10KB Downstream	4.92	1.33	310	7	12	44	44	21	42285	1110	200633	20921
1KB Upstream	4.89	1.35	258	8	11	47	47	22	34254	1162	208664	20921
Sheep ChiP H3K27ac	4.60	1.27	429	11	15	33	33	19.7	61683	991	181235	35266
CDS	4.40	1.25	533	12	16	27	27	18.3	79000	887	163918	226211
1KB Dowsntream	3.83	1.31	237	15	13	50	50	26	32272	1183	210646	20921
Proximal DNase	3.49	1.21	549	16	20	23	23	19.7	83275	871	159643	341731
Proximal All	3.41	1.20	550	17	21	22	22	20	83589	870	159329	346880
Sheep ChIP H3K4me3	3.35	1.30	204	18	14	55	55	29	27758	1216	215160	16098
Proximal TF	3.07	1.21	395	19	19	37	37	25	58686	1025	184232	145993
Proximal H3K9ac	2.66	1.18	438	20	24	32	32	25.3	66546	982	176372	193180
Sheep H3K27ac enhancers	2.42	1.19	326	21	23	42	42	28.7	48669	1094	194249	26492
TssBiv	2.22	1.20	242	23	22	49	49	31.3	35473	1178	207445	27773
Introns	1.76	1.12	612	25	31	17	31	24.3	97893	808	145025	187359
BivFlnk	1.65	1.15	253	26	27	48	48	33.7	38398	1167	204520	37351
TssAFlnk	1.09	1.08	830	32	33	11	33	25.3	137440	590	105478	183766
Intergentic	0.88	1.08	1101	34	34	6	34	24.7	185187	319	57731	22091
UTR	0.78	1.10	121	35	32	59	59	42	18938	1299	223980	14174
Proximal H3K4me3	0.76	1.06	304	36	36	45	45	39	49492	1116	193426	166587
TssA	0.33	1.01	571	40	40	20	40	33.3	97377	849	145541	82792
Distal TF	0.28	1.00	992	42	42	10	42	31.3	169824	428	73094	361496
EnhBiv	0.05	0.93	412	46	46	35	46	42.3	73993	1008	168925	98442
TxWk	0.05	0.93	1025	47	47	9	47	34.3	178784	395	64134	201299
Distal H3K27ac	0.0020558	0.85	1091	52	52	7	52	37	193405	329	49513	632067
Enh no TSSA	0.0003780	0.80	1172	53	54	4	54	37	207708	248	35210	346131
Enh	0.0002924	0.78	1213	55	58	3	58	38.7	214300	207	28618	415857
ReprPC	0.0000954	0.83	533	56	53	27	56	45.3	102255	887	140663	119952
Distal H3K9	0.0000166	0.79	1235	29	30	4	30	21	216964	185	25954	361914
Distal H3K4me1	0.0000005	0.77	572	30	29	17	30	25.3	110238	848	132680	110609
Distal DNase	0.00	0.59	1043	31	31	8	31	23.3	189175	377	53743	550604
Distal_All	0.00	0.58	444	32	32	24	32	29.3	90465	976	152453	374953
Het	0.00	0.62	1311	33	37	1	37	23.7	232361	109	10557	18348

Supplementary Table 9: Overlap of (1420 random regions of 20 kb) regions and list of annotated genomic features based on LOLA [1].

Genomic Feature	Log p-val	Log Odd ratio	support	RnK V	Rnk LO	Rnk Sup	Max Rnk	Mean Rnk	b	c	d	Size
UTR	1.06	1.14	125	1	2	59	59	20.7	18934	1294	223985	14174
Distal H3K9ac	0.99	1.09	1125	2	4	7	7	4.33	189093	294	53826	550604
Distal H3K27ac	0.98	1.09	1149	3	3	6	6	4	193347	270	49572	632067
Distal H3K4me1	0.78	1.06	546	4	7	22	22	11	90363	873	152556	374953
BivFlnk	0.68	1.06	236	5	6	46	46	19	38415	1183	204504	37351
1KB_upstream	0.66	1.06	211	6	5	48	48	19.7	34301	1208	208618	20921
CDS	0.54	1.03	472	8	10	25	25	14.3	79061	947	163858	226211
TssBiv	0.49	1.04	214	10	9	47	47	22	35501	1205	207418	27773
Sheep ChIP H3K4me3	0.44	1.03	167	11	11	55	55	25.7	27795	1252	215124	16098
10KB_upstream	0.44	1.03	271	13	13	43	43	23	45456	1148	197463	20921
TxFlnk	0.44	1.03	207	14	12	49	49	25	34594	1212	208325	34251
TssAFlnk	0.37	1.01	807	16	19	13	19	16	137463	612	105456	183766
Intergenic	0.36	1.01	1085	17	17	8	17	14	185203	334	57716	22091
Introns	0.35	1.01	575	18	20	19	20	19	97930	844	144989	187359
10KB_downstream	0.33	1.01	249	19	22	45	45	28.7	42346	1170	200573	20921
Distal TF	0.33	1.01	994	20	24	10	24	18	169822	425	73097	361496
EnhBiv	0.31	1.00	433	25	25	29	29	26.3	73972	986	168947	98442
Tx	0.19	0.98	448	35	33	28	35	32	77779	971	165140	89599
ReprPC	0.15	0.97	587	39	36	18	39	31	102201	832	140718	119952
TxWk	0.14	0.97	1035	40	39	9	40	29.3	178774	384	64145	201299
1KB_downstream	0.13	0.95	181	41	45	54	54	46.7	32328	1238	210591	20921
Enh only	0.08	0.93	1201	48	50	4	50	34	207679	218	35240	346131
Proximal H3K4me3	0.08	0.94	275	50	49	42	50	47	49521	1144	193398	166587
TssA	0.08	0.95	551	51	47	21	51	39.7	97397	868	145522	82792
Het	0.09	0.95	743	47	42	14	47	34.3	130010	676	112909	119185
Distal DNase	0.08	0.91	1322	52	61	2	61	38.3	227790	97	15129	1373967
EnhG	0.07	0.94	406	53	48	32	53	44.3	72386	1013	170533	100036
Distal All	0.07	0.90	1324	54	62	1	62	39	228129	95	14790	1397221
Sheep ChIP H3K27ac enhancers	0.06	0.93	268	55	56	44	56	51.7	48727	1151	194192	26492
Enh	0.06	0.91	1238	56	58	3	58	39	214275	181	28644	415857
Proximal All	0.05	0.93	467	57	52	26	57	45	83672	952	159247	346880
Proximal Dnase	0.04	0.93	464	58	55	27	58	46.7	83360	955	159559	341731
Sheep ChIP H3K27ac	0.03	0.91	336	62	60	38	62	53.3	61776	1083	181143	35266
Proximal H3K9ac	0.01	0.90	359	64	63	36	64	54.3	66625	1060	176294	193180
Proximal TF	0.01	0.88	311	65	64	41	65	56.7	58770	1108	184149	145993

Supplementary Table 10: Overlap of domestic selective sweeps (635 merged regions) and list of annotated genomic features based on LOLA [1].

Genomic Feature	Log p-val	Log Odd ratio	support	RnK V	Rnk LO	Rnk Sup	Max Rnk	Mean Rnk	b	c	d	Size
1KB_upstream	13.25	2.07	161	1	4	46	46	17	34351	474	209352	20921
1KB_dowsntream	12.82	2.07	153	2	3	49	49	18	32356	482	211347	20921
TxFlnk	12.63	2.03	160	3	5	47	47	18.3	34641	475	209062	34251
Sheep ChIP H3K27ac	12.16	1.82	243	4	16	32	32	17.3	61869	392	181834	35266
10KB upstream	11.77	1.89	192	6	11	41	41	19.3	45535	443	198168	20921
Proximal All	11.21	1.73	302	7	25	21	25	17.7	83837	333	159866	346880
Proximal DNase	11.17	1.73	301	8	27	22	27	19	83523	334	160180	341731
EnhG_original	11.17	1.75	270	9	22	28	28	19.7	72522	365	171181	100036
Proximal H3K9ac	10.83	1.74	252	10	23	30	30	21	66732	383	176971	193180
Sheep ChIP H3K4me3	10.70	2.02	131	12	6	55	55	24.3	27831	504	215872	16098
Tx	10.60	1.71	283	13	30	26	30	23	77944	352	165759	89599
Proximal TF	9.87	1.72	225	15	28	36	36	26.3	58856	410	184847	145993
10KB downstream	9.80	1.81	175	16	18	45	45	26.3	42420	460	201283	20921
CDS	9.75	1.67	283	17	32	26	32	25	79250	352	164453	226211
TssBiv	9.44	1.84	152	18	14	50	50	27.3	35563	483	208140	27773
Sheep ChIP H3K27ac enhancers	8.67	1.71	190	25	31	42	42	32.7	48805	445	194898	26492
TssA	8.41	1.59	327	26	37	18	37	27	97621	308	146082	82792
BivFlnk	7.85	1.72	155	28	29	48	48	35	38496	480	205207	37351
Proximal H3K4me3	7.01	1.61	185	30	36	44	44	36.7	49611	450	194092	166587
UTR	6.58	1.88	87	31	12	58	58	33.7	18972	548	224731	14174
TssAFlnk	5.74	1.47	417	36	41	11	41	29.3	137853	218	105850	183766
Distal TF	4.58	1.46	490	40	42	10	42	30.7	170326	145	73377	361496
Introns	4.17	1.36	304	43	46	19	46	36	98201	331	145502	187359
Enh Biv	2.86	1.29	229	48	49	35	49	44	74176	406	169527	98442
Intergenic	1.98	1.26	509	51	50	7	51	36	185779	126	57924	22091
ReprPC	1.94	1.20	296	52	54	23	54	43	102492	339	141211	119952
TxWk	1.87	1.24	492	53	52	9	53	38	179317	143	64386	201299
Distal H3K27ac.	1.35	1.20	523	54	55	6	55	38.3	193973	112	49730	632067
Het	1.10	1.12	358	55	60	15	60	43.3	130395	277	113308	119185
**Enh excluding TssA**	1.03	1.18	555	56	57	4	57	39	208325	80	35378	346131
Distal DNase	0.80	1.21	602	61	53	1	61	38.3	228510	33	15193	1373967
**Enh**	0.74	1.13	568	62	59	3	62	41.3	214945	67	28758	415857
Distal All	0.71	1.18	602	63	56	1	63	40	228851	33	14852	1397221
Distal H3K9ac	0.66	1.08	503	64	64	8	64	45.3	189715	132	53988	550604
Distal H3K4me1	0.02	0.88	217	65	65	39	65	56.3	90692	418	153011	374953

Supplementary References

1. Sheffield, N. C. & Bock, C. LOLA: enrichment analysis for genomic region sets and regulatory elements in R and Bioconductor. *Bioinformatics* **32,** 587 (2016).
2. Li, Y.-F., Li, H.-F., Zhang, Y.-B. & Wu, J.-M. Novel homozygous PANK2 mutation identified in a consanguineous Chinese pedigree with pantothenate kinase-associated neurodegeneration. *Biomed. Rep.* **5,** 217–220 (2016).
3. Orellana, D. I. *et al.* Coenzyme A corrects pathological defects in human neurons of PANK2-associated neurodegeneration. *EMBO Mol. Med.* **8,** 1197–1211 (2016).
4. Hoekstra, H. The secret of a natural blond. *Nat. Genet.* **46,** 660–661 (2014).
5. Guenther, C. A., Tasic, B., Luo, L., Bedell, M. A. & Kingsley, D. M. A molecular basis for classic blond hair color in Europeans. *Nat. Genet.* **46,** 748–752 (2014).
6. Reissmann, M. & Ludwig, A. Pleiotropic effects of coat colour-associated mutations in humans, mice and other mammals. *Semin. Cell Dev. Biol.* **24,** 576–586 (2013).
7. Meng, Q. *et al.* Identification of growth trait related genes in Large white purebred pig population by GWAS. *Asian-Australas. J. Anim. Sci.* (2016). doi:10.5713/ajas.16.0548
8. Ruchat, S.-M. *et al.* Evidence of interaction between type 2 diabetes susceptibility genes and dietary fat intake for adiposity and glucose homeostasis-related phenotypes. *J. Nutr. Nutr.* **2,** 225–234 (2009).
9. Li, X. *et al.* Variation in IGF2BP2 interacts with adiposity to alter insulin sensitivity in Mexican Americans. *Obes. Silver Spring Md* **17,** 729–736 (2009).
10. Wu, H.-H. *et al.* IGF2BP2 and obesity interaction analysis for type 2 diabetes mellitus in Chinese Han population. *Eur. J. Med. Res.* **19,** 40 (2014).
11. García-Gámez, E. *et al.* Using regulatory and epistatic networks to extend the findings of a genome scan: identifying the gene drivers of pigmentation in merino sheep. *PloS One* **6,** e21158 (2011).

12. Imsland, F. *et al.* Regulatory mutations in TBX3 disrupt asymmetric hair pigmentation that underlies Dun camouflage color in horses. *Nat. Genet.* **48,** 152–158 (2016).

13. Karim, L. *et al.* Variants modulating the expression of a chromosome domain encompassing PLAG1 influence bovine stature. *Nat. Genet.* **43,** 405–413 (2011).

14. Littlejohn, M. *et al.* Genetic variation in PLAG1 associates with early life body weight and peripubertal weight and growth in Bos taurus. *Anim. Genet.* **43,** 591–594 (2012).

15. Nishimura, S. *et al.* Genome-wide association study identified three major QTL for carcass weight including the PLAG1-CHCHD7 QTN for stature in Japanese Black cattle. *BMC Genet.* **13,** 40 (2012).

16. Utsunomiya, Y. T. *et al.* Genome-wide mapping of loci explaining variance in scrotal circumference in Nellore cattle. *PloS One* **9,** e88561 (2014).

17. Våge, D. I. & Boman, I. A. A nonsense mutation in the beta-carotene oxygenase 2 (BCO2) gene is tightly associated with accumulation of carotenoids in adipose tissue in sheep (Ovis aries). *BMC Genet.* **11,** 10 (2010).

18. Tian, R., Pitchford, W. S., Morris, C. A., Cullen, N. G. & Bottema, C. D. K. Genetic variation in the beta, beta-carotene-9’, 10’-dioxygenase gene and association with fat colour in bovine adipose tissue and milk. *Anim. Genet.* **41,** 253–259 (2010).

19. Strychalski, J., Brym, P., Czarnik, U. & Gugołek, A. A novel AAT-deletion mutation in the coding sequence of the BCO2 gene in yellow-fat rabbits. *J. Appl. Genet.* **56,** 535–537 (2015).

20. Scatà, M. C. *et al.* Ovine acyl CoA:diacylglycerol acyltransferase 1- molecular characterization, polymorphisms and association with milk traits. *Anim. Genet.* **40,** 737–742 (2009).

21. Lehnert, K. *et al.* Phenotypic population screen identifies a new mutation in bovine DGAT1 responsible for unsaturated milk fat. *Sci. Rep.* **5,** 8484 (2015).

22. Lindholm-Perry, A. K. *et al.* Association, effects and validation of polymorphisms within the NCAPG - LCORL locus located on BTA6 with feed intake, gain, meat and carcass traits in beef cattle. *BMC Genet.* **12,** 103 (2011).

23. He, S., Zhang, L., Li, W. & Liu, M. BIEC2-808543 SNP in the LCORL Gene is Associated with Body Conformation in the Yili Horse. *Anim. Biotechnol.* **26,** 289–291 (2015).

24. Al-Mamun, H. A. *et al.* Genome-wide association study of body weight in Australian Merino sheep reveals an orthologous region on OAR6 to human and bovine genomic regions affecting height and weight. *Genet. Sel. Evol. GSE* **47,** 66 (2015).

25. Takasuga, A. PLAG1 and NCAPG-LCORL in livestock. *Anim. Sci. J. Nihon Chikusan Gakkaiho* **87,** 159–167 (2016).

26. Braglia, S. *et al.* Comparison of genetic models for analysing the effects of a PvuII polymorphism in the oestrogen receptor 1 (ESR1) gene on prolificacy in an Iberian x Meishan pig population. *Anim. Genet.* **37,** 454–458 (2006).

27. Muñoz, M. *et al.* Non-additive effects of RBP4, ESR1 and IGF2 polymorphisms on litter size at different parities in a Chinese-European porcine line. *Genet. Sel. Evol. GSE* **42,** 23 (2010).

28. Hudson, N. J., Reverter, A., Wang, Y., Greenwood, P. L. & Dalrymple, B. P. Inferring the transcriptional landscape of bovine skeletal muscle by integrating co-expression networks. *PloS One* **4,** e7249 (2009).

29. Tallam, A. *et al.* Gene Regulatory Network Inference of Immunoresponsive Gene 1 (IRG1) Identifies Interferon Regulatory Factor 1 (IRF1) as Its Transcriptional Regulator in Mammalian Macrophages. *PloS One* **11,** e0149050 (2016).

30. Rupp, R. *et al.* A Point Mutation in Suppressor of Cytokine Signalling 2 (Socs2) Increases the Susceptibility to Inflammation of the Mammary Gland while Associated with Higher Body Weight and Size and Higher Milk Production in a Sheep Model. *PLoS Genet.* **11,** e1005629 (2015).

31. Wu, S. *et al.* Genome-wide scans reveal variants at EDAR predominantly affecting hair straightness in Han Chinese and Uyghur populations. *Hum. Genet.* **135,** 1279–1286 (2016).

32. Ji, X.-Y. *et al.* Comparative Transcriptome Analysis Reveals that a Ubiquitin-Mediated Proteolysis Pathway Is Important for Primary and Secondary Hair Follicle Development in Cashmere Goats. *PloS One* **11,** e0156124 (2016).

33. Lu, J. *et al.* Transcript characteristic of myostatin in sheep fibroblasts. *J. Cell. Biochem.* **113,** 2652–2660 (2012).

34. Huang, J. *et al.* Inferring the Skeletal Muscle Developmental Changes of Grazing and Barn-Fed Goats from Gene Expression Data. *J. Agric. Food Chem.* **64,** 6791–6800 (2016).

35. Osterwalder, M. *et al.* HAND2 targets define a network of transcriptional regulators that compartmentalize the early limb bud mesenchyme. *Dev. Cell* **31,** 345–357 (2014).

36. Brown, H. M., Fabre Nys, C., Cognié, J. & Scaramuzzi, R. J. Short oestrous cycles in sheep during anoestrus involve defects in progesterone biosynthesis and luteal neovascularisation. *Reprod. Camb. Engl.* **147,** 357–367 (2014).

37. Kijas, J. W. *et al.* Genome-Wide Analysis of the World's Sheep Breeds Reveals High Levels of Historic Mixture and Strong Recent Selection. *PLOS Biol* **10,** e1001258 (2012).

38. Norris, B. J. & Whan, V. A. A gene duplication affecting expression of the ovine ASIP gene is responsible for white and black sheep. *Genome Res.* **18,** 1282–1293 (2008).

39. Takahashi, K. & Yamanaka, S. Induction of pluripotent stem cells from mouse embryonic and adult fibroblast cultures by defined factors. *Cell* **126,** 663–676 (2006).

40. Tarabeux, J. *et al.* Rare mutations in N-methyl-D-aspartate glutamate receptors in autism spectrum disorders and schizophrenia. *Transl. Psychiatry* **1,** e55 (2011).

41. Haase, B., Jagannathan, V., Rieder, S. & Leeb, T. A novel KIT variant in an Icelandic horse with white-spotted coat colour. *Anim. Genet.* **46,** 466 (2015).

42. Szczerbal, I. *et al.* Ectopic position of duplicated KIT gene in African Nguni cattle, associated with color sidedness, confirms its shared ancestry with the Bos taurus lineage. *Anim. Genet.* (2016). doi:10.1111/age.12495

43. Villar, D. *et al.* Enhancer Evolution across 20 Mammalian Species. *Cell* **160,** 554 (2015).
