# Supplementary material for: Sheep genome functional annotation reveals proximal regulatory elements contributed to the evolution of modern breeds
Source: Nat Commun. 2018 Feb 28;9:859. doi: 10.1038/s41467-017-02809-1 (PMC5830443; doi:10.1038/s41467-017-02809-1)
Supplement: Supplementary file 3 — Description of Additional Supplementary Files [file 41467_2017_2809_MOESM3_ESM.pdf]

Description of Additional Supplementary Files

File Name: Supplementary Data 1
Description: Summary statistics on sequencing and mapping quality per sample

File Name: Supplementary Data 2
Description: Variants Summary statistics per phylo-geographic group and individual

File Name: Supplementary Data 3
Description: Detected Selective sweeps 20 kB in domestic sheep

File Name: Supplementary Data 4
Description: Detected Selective sweeps 20 kB in wild mouflon

File Name: Supplementary Data 5
Description: Detected Selective Sweeps in domestic sheep based on Fst and nucleotide diversity across wild (*Ovis orientalis*) and domestic sheep (*Ovis aries*) (p.adj <0.01) and closest genes to regions

File Name: Supplementary Data 6
Description: Detected Selective Sweeps in mouflon based on Fst and nucleotide diversity across wild (*Ovis orientalis)* and domestic sheep (*Ovis aries*) (p.adj <0.01) and closest genes to regions

File Name: Supplementary Data 7
Description: Gene Ontology enrichment analysis of Biological Process in GREAT

File Name: Supplementary Data 8
Description: Mouse Genome Informatics (MGI) Phenotype Enrichment analysis in GREAT

File Name: Supplementary Data 9
Description: Allele Frequencies for 14 million SNPs MAF>0.05

File Name: Supplementary Data 10
Description: List of missense mutations with ΔAF>0.8
